# Jaboticaba Peel Extract Attenuates Ovariectomy-Induced Bone Loss by Preserving Osteoblast Activity

**DOI:** 10.3390/biology13070526

**Published:** 2024-07-16

**Authors:** Letícia Faustino Adolpho, Maria Paula Oliveira Gomes, Gileade Pereira Freitas, Rayana Longo Bighetti-Trevisan, Jaqueline Isadora Reis Ramos, Gabriela Hernandes Campeoti, Guilherme Crepi Zatta, Adriana Luisa Gonçalves Almeida, Adriana Gadioli Tarone, Mario Roberto Marostica-Junior, Adalberto Luiz Rosa, Marcio Mateus Beloti

**Affiliations:** 1 Bone Research Lab, Ribeirão Preto School of Dentistry, University of São Paulo, Av do Café s/n, Ribeirão Preto 14040-904, SP, Brazil; leticia.adolpho@usp.br (L.F.A.); maria.paula.gomes@usp.br (M.P.O.G.); rayana.bighetti@usp.br (R.L.B.-T.); jaqueline.isadora.ramos@usp.br (J.I.R.R.); gabicampeoti@gmail.com (G.H.C.); guilhermecpz@usp.br (G.C.Z.); aalmeida@forp.usp.br (A.L.G.A.); adalrosa@forp.usp.br (A.L.R.); 2 Department of Oral and Maxillofacial Surgery, School of Dentistry, Federal University of Goiás, Avenida Universitária, s/n—Setor Leste Universitário, Goiânia 74605-020, GO, Brazil; gileade@ufg.br; 3 School of Food Engineering, University of Campinas, Rua Monteiro Lobato 80, Campinas 13083-862, SP, Brazil; dricagt@gmail.com (A.G.T.); mmarosti@unicamp.br (M.R.M.-J.)

**Keywords:** adipocyte, animal model, bone loss, jaboticaba, osteoblast, osteoporosis

## Abstract

**Simple Summary:**

Therapies to prevent osteoporosis are relevant since it is one of the most common non-communicable human diseases in the world and the most prevalent bone disorder in adults. Jaboticaba is a small, round fruit with white pulp and purple peel that concentrates compounds that may have therapeutic applications. Since jaboticaba peel extract (JPE) enhances the activity of osteoblasts (the cells that form bone) under osteoporotic conditions, we hypothesized that JPE prevents the development of osteoporosis induced by the removal of the ovaries. Ovariectomized rats were treated with either JPE (30 mg/kg of body weight) or its vehicle for 90 days, starting 7 days after the ovariectomy. JPE attenuated ovariectomy-induced bone loss due to its ability to prevent the imbalance between osteoblast and adipocyte (the cells that produce bone and fat, respectively) differentiation. These data indicate the potential therapeutic use of JPE to prevent osteoporosis.

**Abstract:**

Therapies to prevent osteoporosis are relevant since it is one of the most common non-communicable human diseases in the world and the most prevalent bone disorder in adults. Since jaboticaba peel extract (JPE) added to the culture medium enhanced the osteogenic potential of mesenchymal stem cells (MSCs) derived from osteoporotic rats, we hypothesized that JPE prevents the development of ovariectomy-induced osteoporosis. Ovariectomized rats were treated with either JPE (30 mg/kg of body weight) or its vehicle for 90 days, starting 7 days after the ovariectomy. Then, the femurs were subjected to microcomputed tomography and histological analyses, and the osteoblast and adipocyte differentiation of MSCs was evaluated. JPE attenuated ovariectomy-induced bone loss, as evidenced by higher bone volume/total volume and trabecular number, along with lower trabecular separation and bone marrow adiposity. These protective effects of JPE on bone tissue are due to its ability to prevent the imbalance between osteoblast and adipocyte differentiation of MSCs, since, compared with MSCs derived from ovariectomized rats treated with vehicle, MSCs treated with JPE exhibited higher gene and protein expression of osteogenic markers and extracellular matrix mineralization, as well as lower gene expression of adipogenic markers. These data highlight the potential therapeutic use of JPE to prevent osteoporosis.

## 1. Introduction

Osteoporosis is a systemic disease characterized by low bone mineral density and disruption of bone microarchitecture, which compromise tissue strength, increasing the risk of fractures. Women after 55 and men after 65 years old are the most affected by fractures resulting from osteoporosis that lead to high levels of morbidity, healthcare costs and deaths [1,2]. Women are more susceptible to osteoporosis due to the decline in estrogen levels during menopause, which can be associated with other factors such as secondary hyperparathyroidism, chronic inflammation and senility [2,3]. On the other hand, men older than 60 in treatment for hip fractures have higher in-hospital mortality than women [4,5]. While aging is associated with an increased bone remodeling rate combined with a negative remodeling balance in women, in men, aging is mostly related to reduced bone formation and low bone turnover [1]. In both cases, osteoporosis-induced bone loss results from an imbalance between bone resorption by osteoclasts and bone formation by osteoblasts [6].

Lifestyle and nutrients affect bone mass; regular exercise and weight loss attenuate the effects of osteoporosis; and calcium and vitamin D are necessary to keep healthy bones and are the first choices to prevent osteoporosis [6,7]. In general, therapies to treat osteoporosis are based on either antiresorptive agents or anabolic drugs, such as bisphosphonates and parathyroid hormone analogues. However, although these agents are effective if correctly prescribed, treatment adherence is challenging mainly due to patients’ perceptions of side effects such as osteonecrosis of the jaw associated with bisphosphonates and a lack of disease information [1,6]. Thus, novel therapies focused on osteoporosis prevention are of relevance in terms of individual well-being and healthcare costs. In this scenario, several plant extracts rich in flavonoids have been investigated for their potential effects on bone tissue [8,9].

Jaboticaba (*Myrciaria jaboticaba* (Vell.) Berg.) is an indigenous Brazilian fruit found in most parts of the country, particularly in the southeast, that has a white pulp and purple peel that concentrate compounds such as phenolic acids and anthocyanins [10,11,12]. Jaboticaba peel extract (JPE) exhibits a protective effect on both weight gain induced by a high-fat diet and obesity-associated insulin resistance, reduces hepatic lipid accumulation and exhibits antioxidant and anti-inflammatory properties [12,13,14,15,16]. Despite the potential health benefits of JPE, its effects on bone tissue have been underexplored. As cyanidin-3-*O*-glucoside (C3G), one of the main bioactive compounds of jaboticaba peel, inhibits osteoclasts and enhances osteoblast differentiation, it is plausible that JPE can modulate the function of bone cells [13,17,18]. Indeed, JPE inhibits lipid accumulation as well as the expression of adipocyte markers such as adiponectin (*Adipoq*), resistin (*Retn*) and adipocyte protein 2 (*aP2*). In addition, it enhances the expression of osteoblast markers such as runt-related transcription factor 2 (*Runx2*) and osteocalcin (*Oc*), alkaline phosphatase (ALP) activity and extracellular matrix mineralization in cultures of bone marrow-derived mesenchymal stem cells (MSCs) [19]. Interestingly, the inhibitory effect of JPE on adipocyte differentiation is more evident in MSCs from healthy rats, while its stimulation of osteoblast differentiation is more pronounced in MSCs from osteoporotic animals [19].

Because of the positive effect of JPE on osteoblast differentiation and the lack of data on the effects of JPE on osteoporotic bones, the hypothesis of this study is that JPE attenuates the development of osteoporosis. To test it, ovariectomized rats were treated with JPE, which attenuated ovariectomy-induced bone loss and reduced the number of adipocytes without affecting osteoclast activity. These findings are due to the effects of JPE on the osteoblast and adipocyte differentiation of MSCs, as MSCs derived from ovariectomized rats treated with JPE were more osteogenic and less adipogenic compared with MSCs derived from ovariectomized rats treated with vehicle.

## 2. Materials and Methods

### 2.1. Ovariectomy and JPE Treatment

The use of animals to conduct this study was approved by the Committee of Ethics in Animal Research of Ribeirão Preto School of Dentistry, University of São Paulo (#2020.1.520.58.8). Sixty-six Sprague Dawley female rats weighing 250 g were subjected to bilateral ovariectomy as described elsewhere [20]. This surgical procedure has proven to be the most popular and efficient method to induce osteoporosis in rats by reducing the levels of estrogenic hormones, mimicking the postmenopausal osteoporosis of adult women. We previously demonstrated that 90 days post-surgery, the femurs of ovariectomized rats exhibited a reduction of 75% in bone mineral density (BMD), 85% in bone volume/total volume (BV/TV), 87% in trabecular number (Tb.N) and an increase of 565% in trabecular separation (Tb.Sp) compared with SHAM animals [19]. Seven days after surgery, the animals started to be treated with either 30 mg of JPE per kg of body weight per day (*n* = 11) or a water vehicle (control, *n* = 10) by oral gavage. Three rats per cage were housed in the animal facility following the Laboratory Animal guidelines and received food and water ad libitum. Rats were euthanized after 90 days of treatment, and the femurs were harvested for microcomputed tomography (µCT), histological analyses and isolation of bone marrow-derived MSCs.

The concentration of JPE and the choice of a preventive approach were based on a pilot study (Supplementary Materials). The pilot study showed that the administration of 3 mg of JPE per kg of body weight (*n* = 11) for 90 days starting 7 days post-ovariectomy slightly improved the evaluated bone morphometric parameters (Figure S1) compared with the control (*n* = 9), suggesting that higher concentrations of JPE could be more effective in preventing osteoporosis development. On the other hand, the administration of 3 mg of JPE per kg of body weight (*n* = 13) for 90 days starting 90 days post-ovariectomy, which means after osteoporosis had been established, did not seem to affect the bone morphometric parameters evaluated (Figure S2) compared with the control (*n* = 12). Thus, we conducted this study using 30 mg of JPE per kg of body weight (10 times higher than the dose used in the pilot study) to evaluate its efficacy in preventing ovariectomy-induced osteoporosis.

### 2.2. Effect of JPE Treatment on Bone Tissue

#### 2.2.1. µCT analysis

The femurs were scanned using the SkyScan 1172 system (Bruker, Kontich, Belgium); 10 μm pixel images were obtained at 60 kV and 165 μA, and the three-dimensional reconstructions were generated using the NRecon software (version 1.6.10.4, Bruker). The region of interest for morphometric analysis started 0.5 mm from the end of the growth plate and ran to 2 mm toward the diaphysis. BV/TV, Tb.N and Tb.Sp were evaluated in the trabecular bone in the region of interest.

#### 2.2.2. Counting of Osteocytes and Adipocytes

After µCT scanning, the femurs were decalcified in 10% ethylenediaminetetraacetic acid (Merck Millipore, Darmstadt, Hesse, Germany), dehydrated, diaphanized, embedded in paraffin (Sigma-Aldrich, St. Louis, MO, USA), cut in 5 µm thick sections and stained with hematoxylin and eosin (Neon, Suzano, SP, Brazil). To evaluate the number of osteocytes and adipocytes in the histological sections, twelve images (magnification: 200×), obtained from the animals treated with either JPE or the vehicle (*n* = 3 per group), were analyzed using a DMLB trinocular microscope for bright field (Leica Microsystems, Wetzlar, Hesse, Germany) coupled with a digital camera DFC3ve00FX and LAS software version 4.1.0 for image acquisition and analysis (Leica Microsystems). The analysis was performed using the ImageJ Software (version 1.50i, NIH, Bethesda, MD, USA), and data were expressed as the number of cells.

#### 2.2.3. Tartrate-Resistant Acid Phosphatase (TRAP) Staining

The osteoclast activity was evaluated by TRAP staining. Histological sections were pre-warmed in TRAP staining solution comprising the basic incubation medium (Sigma-Aldrich) for TRAP detection, fast red violet LB salt (0.6 mg/mL) (Sigma-Aldrich) and naphthol AS-MX phosphate as the substrate (5 μL/mL) (Sigma-Aldrich) at 37 °C for 30 min and counterstained with 0.08% fast green (Sigma-Aldrich) at room temperature for 5 min. To evaluate the TRAP-stained area, five images (magnification: 100×), obtained from the animals treated with either JPE or the vehicle (*n* = 3 per group), were analyzed using a DMLB trinocular microscope for bright field (Leica Microsystems) coupled with a digital camera DFC300FX and LAS software version 4.1.0 for image acquisition and analysis (Leica Microsystems).

### 2.3. Effect of JPE Treatment on Osteoblast and Adipocyte Differentiation of MSCs

#### 2.3.1. MSC Isolation and Osteoblast and Adipocyte Differentiation

MSCs were harvested from the femur bone marrow of animals treated with either JPE or its vehicle, as previously described [21]. Then, the MSCs were cultured in a growth medium composed of minimum essential medium alpha modification (Gibco-Life Technologies, Grand Island, NY, USA), 10% fetal bovine serum (Gibco-Life Technologies), 1% penicillin–streptomycin (Gibco-Life Technologies) and 0.3 μg/mL of fungizone (Gibco-Life Technologies) until subconfluence. The MSCs were maintained at 37 °C in a humidified atmosphere with 5% CO_2_ and 95% atmospheric air, and the medium was replaced every 48 h.

To induce cell differentiation, cells were detached and cultured (2 × 10^4^ cells/well) in 24-well culture plates (Corning Inc., Corning, NY, USA) for up to 21 days in either osteogenic or adipogenic medium. The osteogenic medium was composed of growth medium supplemented with 10^−7^ M dexamethasone (Sigma-Aldrich), 50 μg/mL ascorbic acid (Gibco-Life Technologies) and 7 mM β-glycerophosphate (Sigma-Aldrich). The adipogenic medium was composed of Dulbecco’s modified eagle medium (Gibco-Life Technologies) supplemented with 10% fetal bovine serum (Gibco-Life Technologies), 10^−6^ M dexamethasone (Sigma-Aldrich), 0.5 μM 3-isobutyl-1-methylxanthine (Sigma-Aldrich), 10 μg/mL insulin (Sigma-Aldrich) and 0.1 M indomethacin (Sigma-Aldrich).

#### 2.3.2. Gene Expression of Osteogenic and Adipogenic Markers

On day 7, total RNA of MSCs cultured in osteogenic or adipogenic medium was extracted using Trizol reagent (Invitrogen, Carlsbad, CA, USA), and 1 μg of total RNA from each sample was used to synthesize the complementary DNA (cDNA). Gene expression of the osteoblast markers *Runx2*, *Alp* and *Oc* was evaluated in the osteogenic cultures, and the adipocyte markers peroxisome proliferator-activated receptor-gamma (*Pparγ*), *Adipoq* and *Retn* were evaluated in the adipogenic cultures by real-time quantitative polymerase chain reaction (qRT-PCR). The reactions (*n* = 4) were carried out using 7 μL of Fast SybrGreen Master-Mix (Applied Biosystems, Foster City, CA, USA), 5 μL of cDNA and appropriate primer sequences (Table S1) in a QuantStudio™ 7 Flex system (Applied Biosystems). The gene expression of the osteoblast markers was normalized to glyceraldehyde-3-phosphate dehydrogenase (*Gapdh*) and that of the adipocyte markers to actin beta (*Actb*) because they were the most stable housekeeping genes for each culture. Relative gene expression was calculated using the cycle threshold value and the 2^−ΔΔCt^ method.

#### 2.3.3. Protein Expression of Osteogenic Markers

On day 7, the expression of the RUNX2 and ALP proteins in the MSCs cultured in osteogenic medium was evaluated by Western blot. The primary antibodies antiRUNX2 (1:1000, rabbit monoclonal antibody—8486S—Cell Signaling Technology, Danvers, MA, USA) and antiALP (1:1000, rabbit polyclonal antibody—ab65834—Abcam, Cambridge, UK) were used, followed by incubation with secondary antibody goat anti-rabbit immunoglobulin (Ig)G–horseradish peroxidase (HPR) secondary antibody (1:3000; 7074S, Cell Signaling Technology) for 1 h at 4 °C and 1 h at room temperature, respectively. Protein expression of GAPDH served as a control and was detected using primary antiGAPDH (1:1000; rabbit polyclonal antibody—sc25778—Santa Cruz Biotechnology, Dallas, TX, USA) and secondary antibody goat anti-rabbit IgG-HPR (1:3000—7074S—Cell Signaling Technology), both incubated for 1 h at room temperature. Protein bands were visualized using ClarityTM Western ECL Substrate (Bio-Rad Laboratories, Hercules, CA, USA). RUNX2 and ALP protein expressions were quantified (*n* = 3) using ImageJ Software (version 1.50i, NIH) and normalized to GAPDH.

#### 2.3.4. Extracellular Matrix Mineralization

On day 21, extracellular matrix mineralization of the MSCs cultured in osteogenic medium was detected using 2% alizarin red solution staining (Sigma-Aldrich) as described elsewhere [21]. Macroscopic images were captured using a Canon EOS Digital Rebel digital camera with a 6.3-megapixel sensor and EF100 f/2.8 macro lens (Canon, Tokyo, Japan). Mineralization was quantified as previously described [22]. Absorbance was measured using a μQuant spectrophotometer (BioTek Instruments Inc., Winooski, VT, USA) at 405 nm, and data (*n* = 5) were expressed as absorbance.

#### 2.3.5. Lipidic Accumulation

On day 21, lipid accumulation of the MSCs cultured in adipogenic medium was detected using oil red O staining solution (Sigma-Aldrich) as previously described [21]. Images of lipid accumulation were captured using a microscope eclipse Ti-S coupled to a DS digital camera with Nis Elements BR software (version 5.02, Nikon Corporation, Tokyo, Japan). To quantify lipid accumulation, the staining was extracted with 100% isopropanol (Merck Millipore), and solution absorbance was detected using a μQuant spectrophotometer (BioTek Instruments Inc.) at 500 nm. Data (*n* = 5) were expressed as absorbance.

### 2.4. Statistical Analyses

Comparisons between JPE and the control were performed using Student’s *t*-test, and a *p*-value of ≤ 0.05 was considered statistically significant.

## 3. Results

### 3.1. Effect of JPE Treatment on Bone Tissue

**Figure 1:**
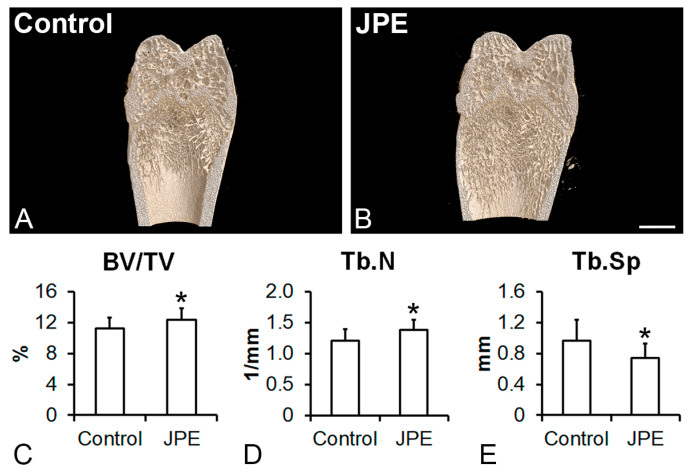
Effect of jaboticaba peel extract (JPE) treatment on bone tissue. Analysis of the femurs by microtomography. Three-dimensional reconstructions of the femur distal epiphysis of ovariectomized rats treated with either vehicle (control) (**A**) or 30 mg of JPE (**B**) per kg of body weight for 90 days starting 7 days post-ovariectomy and morphometric parameters (bone volume/total volume (BV/TV) (**C**), trabecular number (Tb.N) (**D**) and trabecular separation (Tb.Sp) (**E**)). Data are presented as mean ± standard deviation (*n* = 10 for control and *n* = 11 for JPE), and * indicates statistically significant differences between control and JPE (*p* ≤ 0.05). Scale bar (**A**,**B**) = 2 mm.

The three-dimensional reconstructions showed more trabecular bone in the femurs of the rats treated with JPE compared with those of the control rats (Figure 1A,B), which was confirmed by the morphometric parameters. BV/TV (*p* = 0.05) (Figure 1C) and Tb.N (*p* = 0.031) (Figure 1D) were higher in the femurs of the rats treated with JPE compared with the femurs of the control rats, while Tb.Sp (*p* = 0.035) (Figure 1E) was lower in the femurs of the rats treated with JPE compared with those of the control rats.

**Figure 2:**
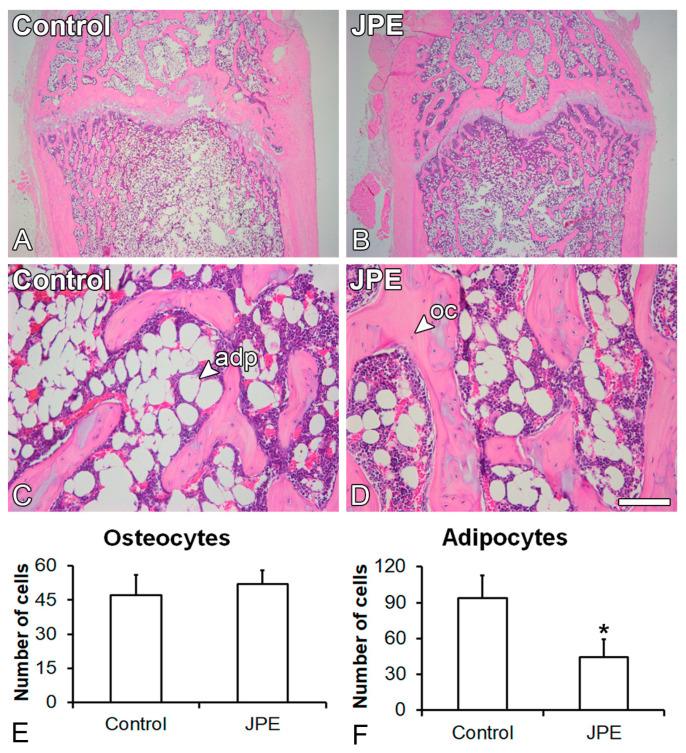
Effect of jaboticaba peel extract (JPE) treatment on bone tissue. Histological analysis of the femurs. Photomicrographs of the femur distal epiphysis of ovariectomized rats treated with either vehicle (control) (**A**,**C**) or 30 mg of JPE (**B**,**D**) per kg of body weight for 90 days starting 7 days post-ovariectomy, stained with hematoxylin and eosin, and number of osteocytes (**E**) and adipocytes (**F**). Data are presented as mean ± standard deviation (*n* = 3), and * indicates statistically significant differences between control and JPE (*p* ≤ 0.05). Scale bar: (**A**,**B**) = 1.25 mm; (**C**,**D**) = 100 μm. adp: adipocyte; oc: osteocyte.

**Figure 3:**
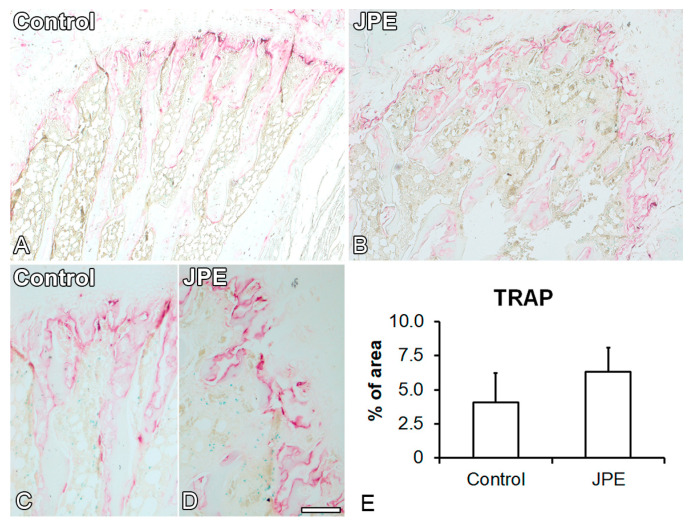
Effect of jaboticaba peel extract (JPE) treatment on bone tissue. Histological analysis of the femurs. Photomicrographs of the femur distal epiphysis of ovariectomized rats treated with either vehicle (control) (**A**,**C**) or 30 mg of JPE (**B**,**D**) per kg of body weight for 90 days starting 7 days post-ovariectomy, stained with tartrate-resistant acid phosphatase (TRAP), and quantification of TRAP staining (**E**). Data are presented as mean ± standard deviation (*n* = 3). Scale bar: (**A**,**B**) = 200 μm; (**C**,**D**) = 100 μm.

The histological analysis showed the presence of trabecular bone and adipose tissue in the medullary spaces of the femurs of both the rats treated with JPE and the control rats (Figure 2A–D). The number of osteocytes was not affected (*p* = 0.488) (Figure 2E), while the number of adipocytes was reduced (*p* = 0.025) (Figure 2F) in the medullary spaces of the femurs of the rats treated with JPE compared with those of the control rats. Osteoclast activity was evidenced by red areas in the femurs of both the rats treated with JPE and the control rats (Figure 3A–D) and was not affected by JPE treatment (*p* = 0.238) (Figure 3E).

### 3.2. Effect of JPE Treatment on Osteoblast Differentiation of MSCs

**Figure 4:**
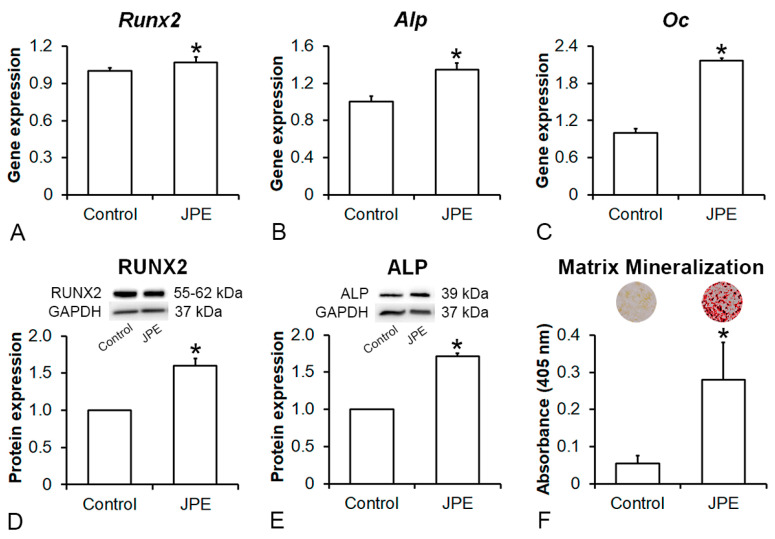
Effect of jaboticaba peel extract (JPE) treatment on osteoblast differentiation of mesenchymal stem cells (MSCs). Gene expression of runt-related transcription factor 2 (*Runx2*) (**A**), alkaline phosphatase (*Alp*) (**B**) and osteocalcin (*Oc*) (**C**) as well as protein expression of RUNX2 (**D**) and ALP (**E**) on day 7 and extracellular matrix mineralization (**F**) on day 21 of bone marrow-derived MSCs obtained from femurs of ovariectomized rats treated with either vehicle (control) or 30 mg of JPE per kg of body weight for 90 days starting 7 days post-ovariectomy, cultured in osteogenic medium. Data are presented as mean ± standard deviation (*n* = 4 for gene expression, *n* = 3 for protein expression and *n* = 5 for extracellular matrix mineralization), and * indicates statistically significant differences between control and JPE (*p * ≤ 0.05).

The MSCs derived from the rats treated with JPE showed higher osteogenic potential than the MSCs derived from the control rats (Figure 4). Gene expression levels of *Runx2* (*p* = 0.035) (Figure 4A), *Alp* (*p* = 0.001) (Figure 4B) and *Oc* (*p* = 0.001) (Figure 4C), as well as protein expression levels (original Western blot images presented in the Supplementary Materials) of RUNX2 (*p* = 0.001) (Figure 4D) and ALP (*p* = 0.001) (Figure 4E) and extracellular matrix mineralization (*p* = 0.001) (Figure 4F) were higher in the MSCs derived from the rats treated with JPE compared with the MSCs derived from the control rats.

### 3.3. Effect of JPE Treatment on Adipocyte Differentiation of MSCs

**Figure 5:**
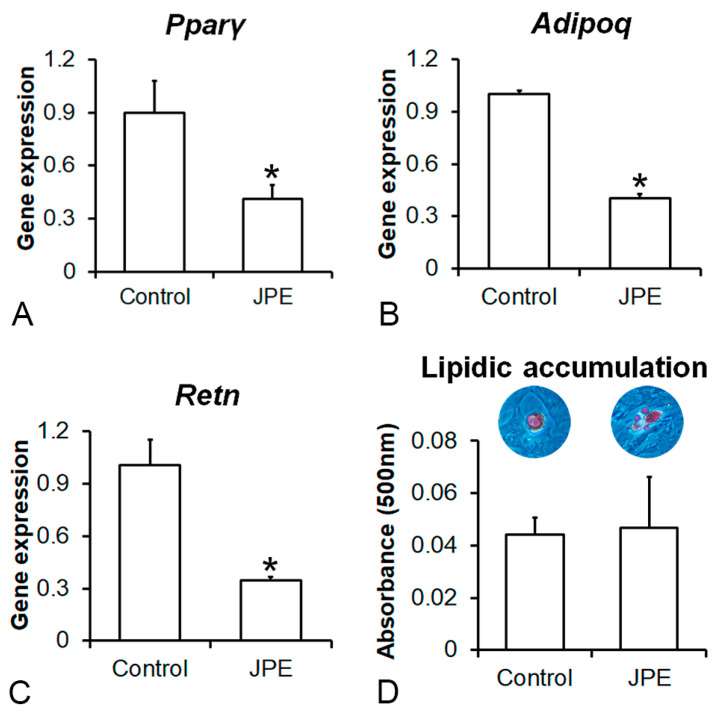
Effect of jaboticaba peel extract (JPE) treatment on adipocyte differentiation of mesenchymal stem cells (MSCs). Gene expression of peroxisome proliferator-activated receptor-gamma (*Pparγ*) (**A**), adiponectin (*Adipoq*) (**B**) and resistin (*Retn*) (**C**) on day 7 as well as lipidic accumulation (**D**) on day 21 of bone marrow-derived MSCs obtained from femurs of ovariectomized rats treated with either vehicle (control) or 30 mg of JPE per kg of body weight for 90 days starting 7 days post-ovariectomy, cultured in adipogenic medium. Data are presented as mean ± standard deviation (*n* = 4 for gene expression and *n* = 5 for lipidic accumulation), and * indicates statistically significant differences between control and JPE (*p* ≤ 0.05).

In general, the MSCs derived from the rats treated with JPE showed lower adipogenic potential than the MSCs derived from the control rats (Figure 5). Gene expression levels of *Pparγ* (*p* = 0.005) (Figure 5A), *Adipoq* (*p* = 0.001) (Figure 5B) and *Retn* (*p* = 0.001) (Figure 5C) were lower in the MSCs derived from the rats treated with JPE compared with the MSCs derived from the control rats, while lipid accumulation was not affected (*p* = 0.783) (Figure 5D) by JPE treatment.

## 4. Discussion

The prevention of osteoporosis development is a major challenge in the medical and pharmacological fields, as it is nowadays one of the most common non-communicable human diseases in the world and the most prevalent bone disease in adults [23]. Considering the relevance of therapies to prevent osteoporosis, this study demonstrated that JPE attenuated ovariectomy-induced bone loss by preserving osteoblast activity. These results rely on the capacity of JPE to protect MSCs from the imbalance between osteoblast and adipocyte differentiation induced by osteoporosis.

The reproducibility of the JPE extraction method was previously confirmed by another study, which also showed the presence of C3G (13.21 ± 0.08 mg/g of dry extract and 6.93 ± 0.04 mg/g of dry jaboticaba peel) and delphinidin-3-glucoside (1.67 ± 0.05 mg/g of dry extract and 0.88 + 0.03 mg/g of dry jaboticaba peel) [24]. The only study reporting the use of JPE in bone cells was conducted using an in vitro model of differentiation of MSCs into osteoblasts, with direct addition of the extract to the culture medium. Thus, the concentration of JPE used in the present study was based on the reduction in hepatic fat accumulation in rats with a diet supplemented with 2% JPE, which corresponds to approximately 0.3 mg of total phenolic compounds per kg of body weight per day [13,19]. Thus, to test the effect of JPE on bone tissue under osteoporotic conditions, either during development or settled disease, rats were treated daily with 3 mg of JPE per kg of body weight, starting either on day 7 or on day 90 post-ovariectomy. This experimental design aimed to demonstrate whether JPE may prevent or restore ovariectomy-induced bone loss. We did not use SHAM animals because we already demonstrated that 90 days post-surgery, the femurs of the ovariectomized rats exhibited a dramatic reduction in BMD, BV/TV and Tb.N and an increase in Tb.Sp compared with SHAM [19]. Additionally, from a therapeutic point of view, JPE would be used to prevent osteoporosis, which represents a challenge in translating our data to clinical applications in terms of selecting target patients. Although no effect was observed in bone tissue irrespective of the period of treatment, a tendency towards improvement in the bone morphometric parameters was noticed with JPE starting 7 days post-ovariectomy, suggesting that higher concentrations of JPE may protect bone tissue from the harmful effects of ovariectomy but could not be useful to treat established osteoporosis. In agreement with this, the purified extract of blueberry, whose polyphenol content is comparable to that of JPE, did not improve the bone mineral density or mechanical properties of osteoporotic bones in ovariectomized rats [25].

These findings guided the next set of experiments that were conducted with rats treated with 30 mg of JPE, which contains approximately 0.40 mg of C3G per kg of body weight daily, starting from day 7 post-ovariectomy. The three-dimensional reconstructions and morphometric parameters generated by the μCT of the femurs and the histological sections clearly showed that JPE inhibited the development of osteoporosis induced by ovariectomy, attenuating bone loss. Based on these promising results, other concentrations of JPE should be tested to find a possible more effective dose to prevent bone loss without exhibiting side effects. Our findings are corroborated by the protective effect of powdered, freeze-dried blackberry—which, similarly to JPE, is a rich source of C3G—against the loss of tibial trabecular number and trabecular bone volume. This may be related to C3G’s antioxidant properties since oxidative stress is associated with osteoporosis-induced bone fragility [26,27,28]. Although both the BV/TV and Tb.N of the femurs of the animals treated with JPE were higher and Tb.Sp was lower than that of the animals treated with vehicle, neither the number of osteocytes nor TRAP activity was affected by JPE. These findings suggest that JPE favored osteoblast differentiation and activity to preserve the bone mass and architecture of ovariectomized rats without interfering with osteoblast proliferation and, consequently, the number of osteocytes and osteoclast activity. The cellular mechanisms that drive this bone tissue response may involve inhibition of the nuclear factor kappa-B (NF-κB) and activation of the sirtuin type 1 deacetylase (Sirt1) by polyphenols, which thereby induce cell differentiation and bone formation [29,30,31,32]. These data support our results, since *Sirt1* gene expression was higher in the MSCs derived from the rats treated with JPE compared with the MSCs derived from the control rats under osteogenic conditions (Figure S3A).

In addition to bone loss and fragility, previous studies have shown that ovariectomized rats and mice, as well as postmenopausal women, have increased bone marrow adiposity [9,33,34]. Our data showed that JPE significantly reduced the number of adipocytes in the bone marrow of ovariectomized rat femurs. In keeping with this, different types and concentrations of jaboticaba peel and seed extracts reduced weight gain and liver steatosis and improved insulin sensitivity. This may be due to their ability to modulate the inflammatory response associated with obesity by downregulating the expression of tumor necrosis factor alpha (TNF-α) and interleukin 6 [13,15,35]. Indeed, phenolic compounds have been shown to reduce weight gain and liver fat accumulation by inhibiting oxidative stress and inflammation in rats and mice fed a high-fat diet [36,37]. Our results are corroborated by these findings, since *Tnf-α* gene expression was lower in the MSCs derived from the rats treated with JPE compared with the MSCs derived from the control rats when cultured in adipogenic medium (Figure S3B).

Osteoporosis disrupts the fate decision of MSCs in favor of adipocytes at the expense of osteoblast differentiation [38,39,40]. We previously demonstrated that JPE inhibited adipocyte and increased osteoblast differentiation of MSCs derived from healthy and osteoporotic rats when the culture media were directly supplemented with the extract [19]. Interestingly, MSCs derived from ovariectomized rats seem to keep the memory of the positive impact of JPE in preserving the balance between osteoblast and adipocyte differentiation, since the animals treated with JPE generated MSCs with higher osteogenic and lower adipogenic potential than the MSCs derived from rats treated with vehicle. These findings corroborate our in vivo results in terms of the reduction in bone loss and bone marrow fat accumulation induced by JPE.

## 5. Conclusions

We demonstrated that JPE attenuated ovariectomy-induced bone loss and bone marrow adiposity without affecting osteoclast activity. These protective effects on bone tissue are due to the ability of JPE to preserve the balance between osteoblast and adipocyte differentiation of MSCs, as the MSCs derived from ovariectomized rats treated with JPE exhibited higher osteogenic and lower adipogenic potential compared with the MSCs derived from ovariectomized rats treated with vehicle. Together, these data, associated with the high availability and low cost of fruits that are rich sources of compounds such as phenolic acids and anthocyanins, pave the way for the development of novel and affordable therapies to prevent osteoporosis.

## Data Availability

The datasets used and analyzed in the current study are available from the corresponding author upon reasonable request.

## Supplementary Materials

The supporting information can be downloaded at https://www.mdpi.com/article/10.3390/biology13070526/s1. Supplementary Materials: Figures S1 and S2: Effect of jaboticaba peel extract (JPE) treatment on bone tissue. Analysis of the femurs by microtomography; Figure S3: Effect of jaboticaba peel extract (JPE) treatment on gene expression of sirtuin type 1 deacetylase (*Sirt1*) and tumor necrosis factor alpha (*Tnf-α*) of mesenchymal stem cells (MSCs); Figure S4: Original images of Western blot; Table S1: Primer sequences for qRT-PCR.
