# Supplementary material for: Jaboticaba Peel Extract Attenuates Ovariectomy-Induced Bone Loss by Preserving Osteoblast Activity
Source: Biology (Basel). 2024 Jul 16;13(7):526. doi: 10.3390/biology13070526 (PMC11273516; doi:10.3390/biology13070526)
Supplement: Supplementary file 1 [file biology-13-00526-s001.zip › biology-3085144-supplementary.pdf]

Supplementary material

Jaboticaba Peel Extract Attenuates Ovariectomy-Induced Bone Loss by Preserving Osteoblast Activity

Letícia Faustino Adolpho [1], Maria Paula Oliveira Gomes [1], Gileade Pereira Freitas [2], Rayana Longo Bighetti-Trevisan [1], Jaqueline Isadora Reis Ramos [1], Gabriela Hernandes Campeoti [1], Guilherme Crepi Zatta [1], Adriana Luisa Gonçalves Almeida [1], Adriana Gadioli Tarone [3], Mario Roberto Marostica-Junior [3], Adalberto Luiz Rosa [1] and Marcio Mateus Beloti [1,*]

1 Bone Research Lab, Ribeirão Preto School of Dentistry, University of São Paulo, Av do Café s/n, Ribeirão Preto 14040-904, SP, Brazil; leticia.adolpho@usp.br (L.F.A.); maria.paula.gomes@usp.br (M.P.O.G.); rayana.bighetti@usp.br (R.L.B.-T.); jaqueline.isadora.ramos@usp.br (J.I.R.R.); gabicampeoti@gmail.com (G.H.C.); guilhermecpz@usp.br (G.C.Z.); aalmeida@forp.usp.br (A.L.G.A.); adalrosa@forp.usp.br (A.L.R.)
2 Department of Oral and Maxillofacial Surgery, School of Dentistry, Federal University of Goiás, Avenida Universitária, s/n—Setor Leste Universitário, Goiânia 74605-020, GO, Brazil; gileade@ufg.br
3 School of Food Engineering, University of Campinas, Rua Monteiro Lobato 80, Campinas 13083-862, SP, Brazil; dricagt@gmail.com (A.G.T.); mmarosti@unicamp.br (M.R.M.-J.)

Leticia Faustino Adolpho and Maria Paula Oliveira Gomes contributed equally to the study.

Corresponding Author:
Marcio Mateus Beloti (ORCID: 0000-0003-0149-7189)
Bone Research Lab, Ribeirão Preto School of Dentistry, University of São Paulo
Av do Café, s/n, 14040-904, Ribeirão Preto, SP, Brazil
Tel.: +55 16 3315 4785
E-mail: mmbeloti@usp.br

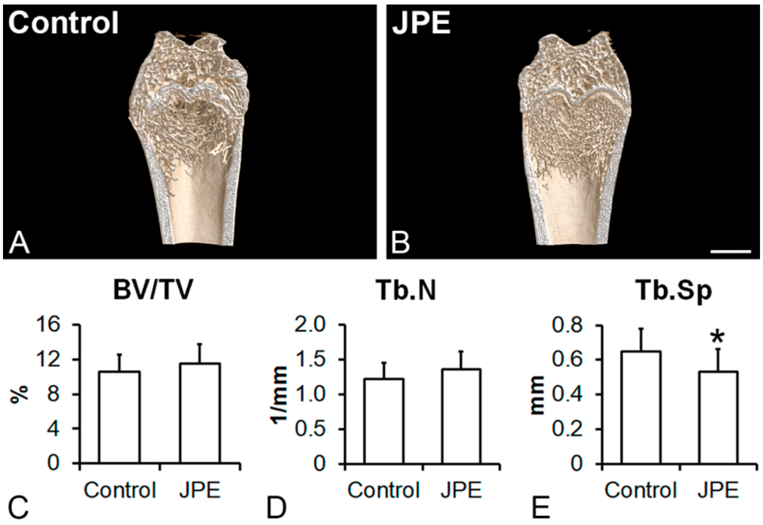

Figure S1. Effect of jaboticaba peel extract (JPE) treatment on bone tissue. Analysis of the femurs by microtomography. Three-dimensional reconstructions of the femur distal epiphysis of ovariectomized rats treated with either vehicle (Control) (**A**) or 3 mg of JPE (**B**) per kg of body weight for 90 days starting 7 days post-ovariectomy, and morphometric parameters bone volume/total volume (BV/TV) ($p = 0.216$) (**C**), trabecular number (Tb.N) ($p = 0.153$) (D) and trabecular separation (Tb.Sp) ($p = 0.039$) (**E**). Data are presented as mean ± standard deviation ($n = 9$ for Control and $n = 11$ for JPE) and * indicates statistically significant differences between Control and JPE ($p \leq 0.05$). Scale bar (A and B) = 2 mm.

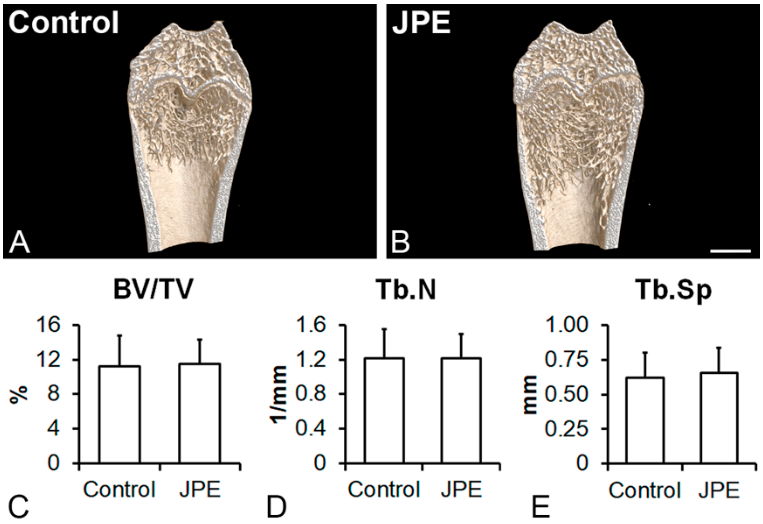

Figure S2. Effect of jaboticaba peel extract (JPE) treatment on bone tissue. Analysis of the femurs by microtomography. Three-dimensional reconstructions of the femur distal epiphysis of ovariectomized rats treated with either vehicle (Control) (**A**) or 3 mg of JPE (**B**) per kg of body weight for 90 days starting 90 days post-ovariectomy, and morphometric parameters bone volume/total volume (BV/TV) ($p = 0.456$) (**C**), trabecular number (Tb.N) ($p = 0.459$) (**D**) and trabecular separation (Tb.Sp) ($p = 0.977$) (**E**). Data are presented as mean ± standard deviation ($n = 12$ for Control and $n = 13$ for JPE). Scale bar (A and B) = 2 mm.

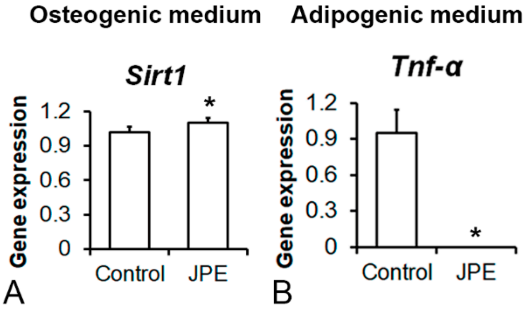

Figure S3. Effect of jaboticaba peel extract (JPE) treatment on gene expression of sirtuin type 1 deacetylase (*Sirt1*) and tumor necrosis factor alpha (*Tnf-α*) of mesenchymal stem cells (MSCs). Gene expression of *Sirt1* ($p = 0.043$) (**A**) and *Tnf-α* ($p = 0.001$) (**B**) on day 7 of bone marrow-derived MSCs obtained from femurs of ovariectomized rats treated with either vehicle (Control) or 30 mg of JPE per kg of body weight for 90 days starting 7 days post-ovariectomy, cultured in either osteogenic (A) or adipogenic (B) medium. Data are presented as mean ± standard deviation (n = 4) and * indicates statistically significant differences between Control and JPE ($p \leq 0.05$).

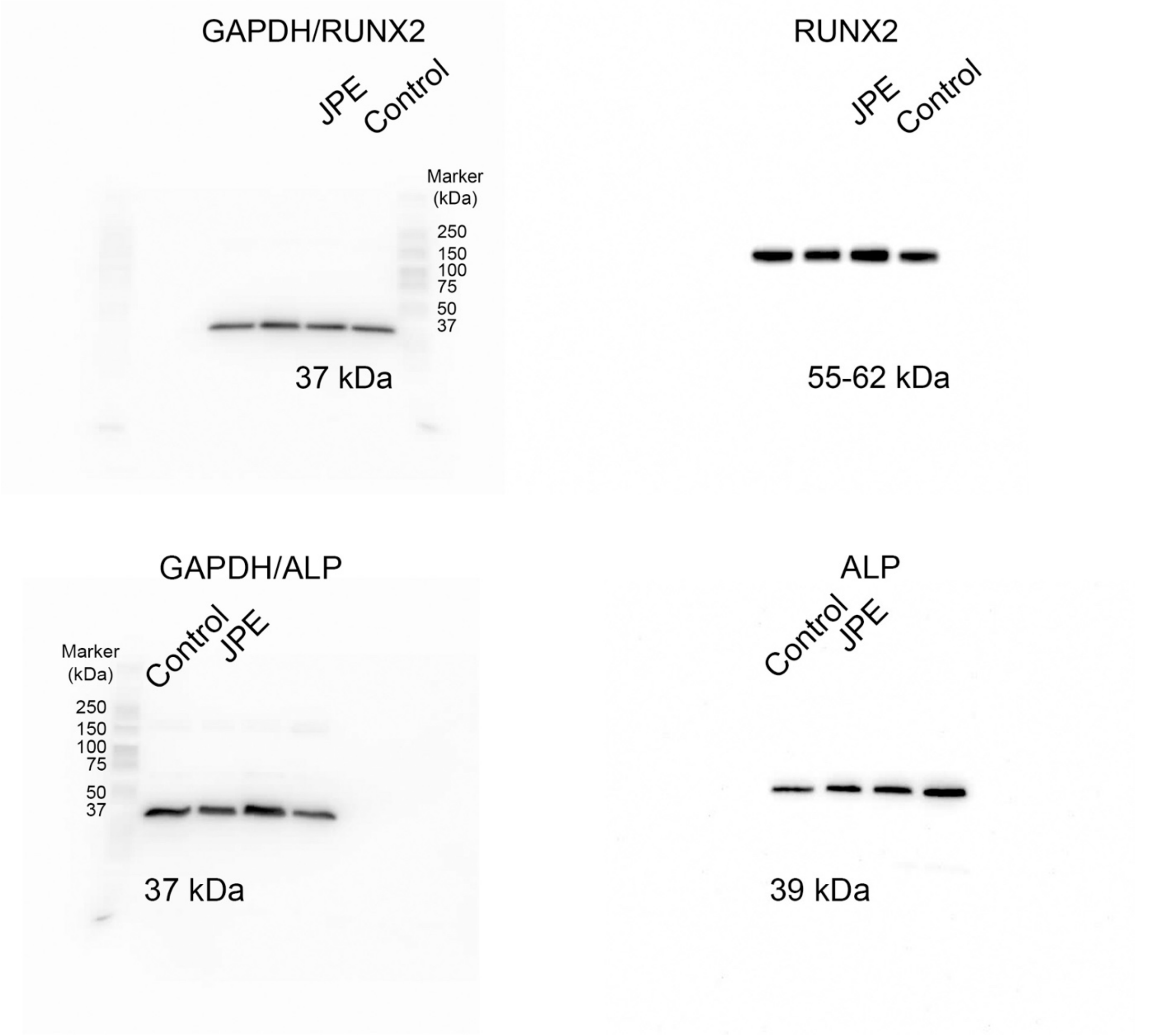

Figure S4 Original images of western blot

Table S1. Primer sequences for qRT-PCR

Target gene	Forward	Reverse
Runx2	CGTATTTCAGATGATGACACTGCC	AAATGCCTGGGAACTGCCTG
Alp	TACTGCTGATCACTCCCACG	ACCGTCCACCACCTTGTAAC
Oc	GCATTCTGCCTCTCTGACCTG	CCGGAGTCTATTCACCACCTTAC
Pparγ	ACTGCCTATGAGCACTTCACA	GGGAGTGGTCATCCATCACAG
Adipoq	GGAAACTTGTGCAGGTTGGAT	GGTCACCCTTAGGACCAAGAA
Retn	ATGGATGAAGCCATCAGCAAGA	GGAGCAGCTAGTGACGGTTG
Sirt1	GGACAGTTCCAGCCATCTCT	TGTTGCAAAGGAACCATGACAC
Tnf-α	CCCAACAAGGAGGAGAAGTTCC	CTCCGCTTGGTGGTTTGCTA
Actb	AACCCTAAGGCCAACCGTG	CATACAGGGACAACACAGCCT
Gapdh	GACAACTTTGGCATCGTGGAA	AGGGATGATGTTCTGGGCTG

Runx2: runt-related transcription factor 2; *Alp*: alkaline phosphatase; *Oc*: osteocalcin; *Pparγ*: peroxisome proliferator-activated receptor-gamma; *Adipoq*: adiponectin; *Retn*: resistin; *Sirt1*: sirtuin type 1 deacetylase; *Tnf-α*: tumor necrosis factor alpha; *Actb*: actin beta; *Gapdh*: glyceraldehyde-3-phosphate dehydrogenase
